# MicroRNA mmu-miR-511-5p: A promising Diagnostic Biomarker in Experimental Toxoplasmosis Using Different Strains and Infective Doses in Mice with Different Immune States Before and After Treatment

**DOI:** 10.1007/s11686-024-00851-w

**Published:** 2024-05-14

**Authors:** Rasha Fadly Mady, Mona Mohamed El-Temsahy, Yasmine Amr Issa, Aya Saied Zaghloul, Safaa Ibrahim Khedr

**Affiliations:** 1 Department of Medical Parasitology, Faculty of Medicine, Alexandria University, 2nd Floor, El Mowasah Medical and Educational Complex, Alexandria, Egypt; 2 Department of Medical Biochemistry, Faculty of Medicine, Alexandria University, Alexandria, Egypt; 3 Medical Biochemistry, College of Medicine, Arab Academy of Science, Technology and Maritime transport, New Alamein campus, Egypt

**Keywords:** *T. gondii*, ME49 strain, RH strain, mmu-miR-511-5p, Spiramycin, Immunosuppression

## Abstract

**Purpose:**

Searching for a novel early diagnostic biomarker for toxoplasmosis, real-time-PCR was currently used to measure the serum mmu-miR-511-5p level in male Swiss-albino mice infected with either; ME49 or RH *Toxoplasma gondii* (*T. gondii*) strains.

**Methods:**

Three mice groups were used; (GI) constituted the non-infected control group, while (GII) and (GIII) were experimentally infected with ME49 or RH strains, respectively. GII mice were orally infected using 10 or 20 ME49 cysts (ME-10 and ME-20), both were subdivided into; non-treated (ME-10-NT and ME-20-NT) and were further subdivided into; *immunocompetent* (ME-10-IC and ME-20-IC) [euthanized 3-days, 1, 2, 6 or 8-weeks post-infection (PI)], and *immunosuppressed* using two Endoxan^®^ injections (ME-10-IS and ME-20-IS) [euthanized 6- or 8-weeks PI], and spiramycin-treated (ME-10-SP and ME-20-SP) that received daily spiramycin, for one-week before euthanasia. GIII mice individually received 2500 intraperitoneal RH strain tachyzoites, then, were subdivided into; non-treated (RH-NT) [euthanized 3 or 5-days PI], and spiramycin-treated (RH-SP) that were euthanized 5 or 10-days PI (refer to the graphical abstract).

**Results:**

Revealed significant upregulation of mmu-miR-511-5p in GII, one-week PI, with gradually increased expression, reaching its maximum 8-weeks PI, especially in ME-20-NT group that received the higher infective dose. Immunosuppression increased the upregulation. Contrarily, treatment caused significant downregulation. GIII recorded significant upregulation 3-days PI, yet, treatment significantly decreased this expression.

**Conclusion:**

Serum mmu-miR-511-5p is a sensitive biomarker for early diagnosis of ME49 and RH infection (as early as one-week and 3-days, respectively), and its expression varies according to *T. gondii* infective dose, duration of infection, spiramycin-treatment and host immune status.

**Graphical abstract:**

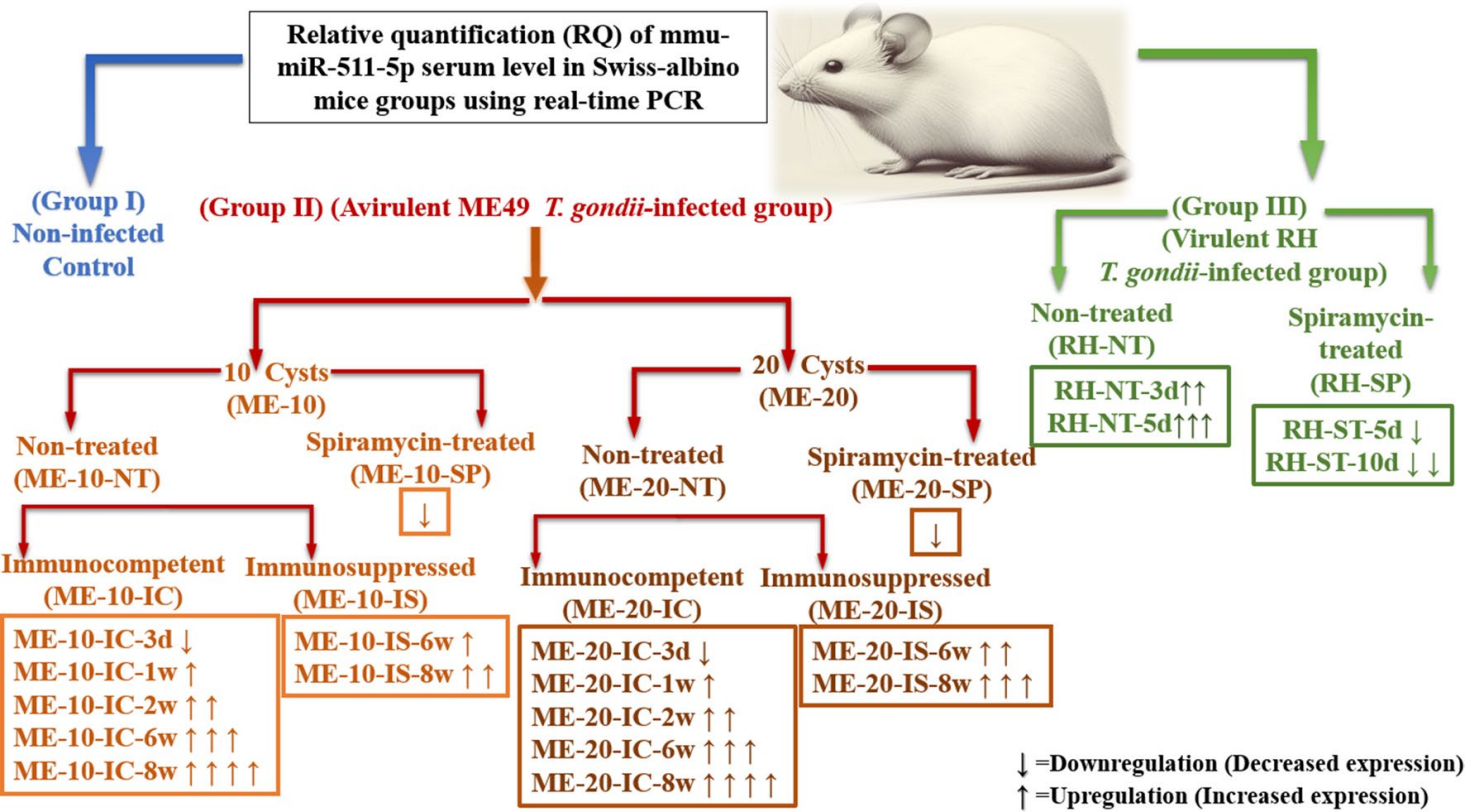

**Supplementary Information:**

The online version contains supplementary material available at 10.1007/s11686-024-00851-w.

## Introduction

*Toxoplasma gondii* is an obligate intracellular protozoan parasite that infects almost all the warm-blooded animals and about 30–50% of the world’s human population [1, 2].

Caused by *T. gondii*, toxoplasmosis is presented by wide spectrum of clinical presentations, based on; inoculum size, organism virulence, and host immunological status, ranging from asymptomatic or flu-like symptoms in immunocompetent patients, up to encephalitis in immunocompromised patients [1, 3, 4]. Moreover, reactivation of latent toxoplasmosis in patients with immunodeficiencies can result in high mortality rates [1, 5].

Classical serological techniques for detection of *T. gondii* antigens or antibodies are the commonly used for diagnosis nowadays. However, several limitations confront them, including; absence of parasite-specific antibodies during the early stages of infection, particularly in immunocompromised patients who fail to produce significant titers of specific antibodies. Moreover, they usually remain positive even after successful treatment, so, they are of low benefit for early detection, and cannot assess treatment [6–8].

Monataya *et al*. (2002) described the limitations in diagnostic techniques for *T. gondii*. They stated that IgG antibodies usually appear within 2 weeks of acquisition of the infection, peak within 1–2 months, decline at various rates, and usually persist for life. Thus, it can’t detect very early infection nor differentiate between new and old infections [9]. As for IgM antibodies, although the commercial test kits measuring them are widely distributed, these tests often have low specificity, and their results are frequently misinterpreted [10]. Regarding IgA antibodies, they may be detected in the sera of acutely infected adults and congenitally infected infants, using ELISA, and they may persist for more than a year [11]. For this reason, they are of little additional assistance for diagnosis of acute infections in adults.

The current diagnostic tools also involve histopathological examination that entails tissue biopsies [12], which can be cumbersome for the patients.

Moving on to molecular tests for toxoplasmosis diagnosis, although they are becoming more frequently applied, yet, they are still controversial, and there is no consensus about the best method or target to be used. In addition, *T. gondii* DNA concentration during chronic infection is usually undetectable, even when molecular sensitivity tests are performed [12]. As a result, diagnosis of *T. gondii* infection is still difficult and the development of alternative methods with higher specificity and sensitivity is crucial [13].

MicroRNAs (miRNAs) are endogenous 18–25 nucleotide non-coding RNA molecules that have fundamental roles in regulating immune response outcomes in eukaryotes, through the miRNA induced silencing complex. Mature miRNAs can directly affect the expression of thousands of genes, by inhibiting protein expression via translational repression and/or mRNA degradation [14, 15].

MiRNAs are stable enough to be detected in the serum, plasma and tissues. Circulating miRNAs are protected from the endogenous RNAse activity [16]. Thus, they have been recognized as appealing biochemical markers for many ailments, including infectious diseases [17–21].

In their review, Judice *et al*. (2016) reported that, many Apicomplexan parasites would alter their host miRNA expression [22]. One of them is, *T. gondii* parasite, which was reported in an earlier study to cause dysregulation in the plasma levels of multiple miRNAs in *T. gondii* infected mice [23]. Among these miRNAs, three miRNAs; (mmu-miR-712-3p, mmu-miR-511-5p, and mmu-miR-217-5p) kept the induced expression in the parasitized mice with both; RH or ME49 strains of *T. gondii* [23].

Because it was not thoroughly investigated in the literature, mmu-miR-511-5p was chosen by the current work’s authors to further assess its role as a tool for early diagnosis of toxoplasmosis and for follow up of its therapy. This was done by monitoring the marker level in the sera of immunocompetent and immunosuppressed Swiss-albino mice experimentally infected with the avirulent (ME49) or the virulent (RH) *T. gondii* strains.

## Materials and Methods

###  Maintenance of *Toxoplasma gondii*

Both avirulent ME49 and virulent RH HXGPRT (-) *T. gondii* strains were maintained in Swiss-albino mice in the Medical Parasitology Department, Alexandria Faculty of Medicine.

For infection by avirulent ME49 strain, cysts were obtained from the brains of infected mice 8 to 15-weeks post-infection (PI). These brains were homogenized in sterile phosphate buffered saline (PBS) (pH = 7.4), Then cysts were counted in the homogenate, and mice were orally infected with 10 or 20 cysts according to their groups [24].

As regards virulent RH HXGPRT(-) strain, it was propagated by serial intraperitoneal passages of tachyzoites in the mice. Tachyzoites were harvested from mice peritoneal lavage on the 5th day PI (dpi) and were kept in sterile PBS at 4^○^C until used [25]. Each mouse was intraperitoneally infected with 2500 tachyzoites [21].

### Grouping of Experimental Animals

**Fig. 1:**
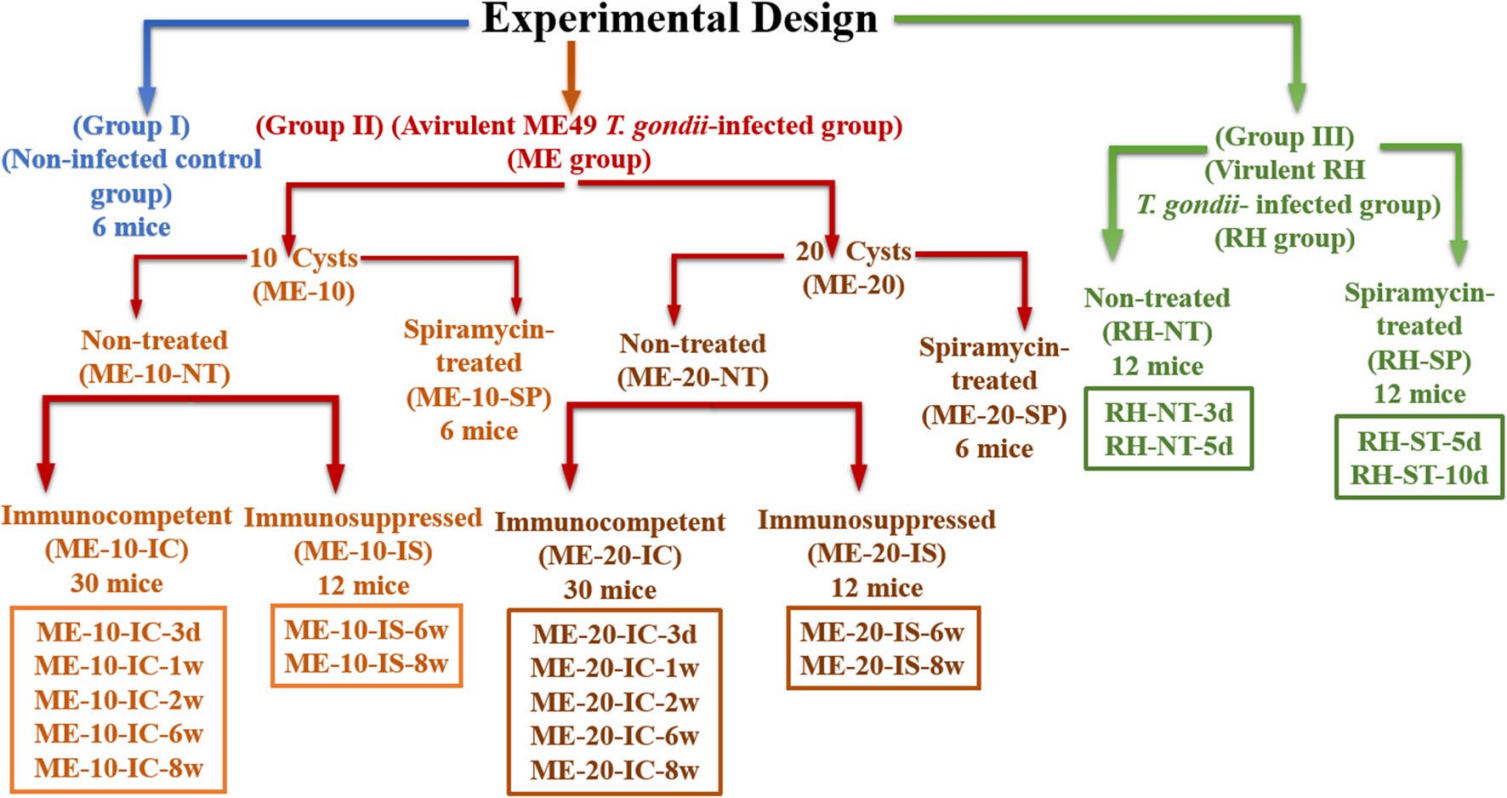
Experimental design used in the current study

The study was performed using 126 male Swiss-albino mice, 4–6 weeks old, weighing 20–25 g each. Animals were housed in dry, clean, well-ventilated cages, with free access to water and food. They were kept in the animal house, in the Medical Parasitology Department, Faculty of Medicine, Alexandria University, according to the national guidelines of animal experimentation. The followed experimental design is shown in Fig. 1. Experimental mice were divided into three groups as follows:

#### Group I (Non-Infected Control Group)

Six non-infected mice were used as control.

#### Group II (ME Group)

Ninety-six mice were infected with the avirulent ME49 strain, and were divided equally, according to the dose of infection, into two subgroups:

##### Subgroup ME-10

Forty-eight mice were orally infected with 10 cysts of ME49 strain and were further subdivided into spiramycin-treated (ME-10-SP) and non-treated (ME-10-NT).

ME-10-SP subgroup included 6 mice, that received oral spiramycin (Rovac^®^), 8-weeks PI in a dose of 400 mg/kg, daily for 7-days [26]. One-day after the end of treatment, mice were anesthetized with ether inhalation, then, they were euthanized using cervical dislocation.

While ME-10-NT subgroup included 42 mice, that were further subdivided into immunocompetent (ME-10-IC) and immunosuppressed (ME-10-IS). ME-10-IC included 30 mice which were subgrouped according to the time of euthanasia, into 5 subgroups; ME-10-IC-3d, ME-10-IC-1w, ME-10-IC-2w, ME-10-IC-6w, and ME-10-IC-8w. Each subgroup contained 6 mice that were euthanized; 3-days, 1, 2, 6, and 8-weeks PI, respectively.

Regarding, ME-10-IS subgroup, it included 12 mice, which were immunosuppressed using intra-peritoneal injection of cyclophosphamide (Endoxan^®^) in 2 doses of 70 mg/kg each, 1-week apart, starting 4-weeks PI [21]. They were divided into; ME-10-IS-6w and ME-10-IS-8w, that were euthanized 6 and 8-weeks PI, respectively.

##### Subgroup ME-20

This included 48 mice, that were orally infected with 20 ME49 cysts and were subdivided into spiramycin-treated (ME-20-SP) and non-treated (ME-20-NT).

ME-20-SP included 6 mice, that received 400 mg/kg of oral spiramycin, 8-weeks PI, daily for one-week [26], then, they were euthanized one-day after the end of treatment.

While, ME-20-NT subgroup included 42 mice that were further subdivided into immunocompetent (ME-20-IC) and immunosuppressed (ME-20-IS). ME-20-IC included 30 mice which were divided according to the time of euthanasia into 5 subgroups; ME-20-IC-3d, ME-20-IC-1w, ME-20-IC-2w, ME-20-IC-6w, and ME-20-IC-8w. Each subgroup contained 6 mice.

Regarding ME-20-IS, it included 12 immunosuppressed mice according to the method of Mogahed *et al*. (2018) [21]. They were equally subgrouped into ME-20-IS-6w and ME-20-IS-8w.

#### Group III (RH Group)

This included 24 mice, which were, individually, infected by 2500 tachyzoites of the RH virulent strain, then they were equally subdivided into non-treated (RH-NT) and spiramycin-treated (RH-SP) subgroups.

RH-NT subgroup included 12 mice which were equally subgrouped into; RH-NT-3d and RH-NT-5d, where mice were euthanized 3 or 5-dpi.

While, RH-SP included 12 mice, which received a daily dose of 400 mg/kg of oral spiramycin, starting on the 1st day of infection. Then, they were equally subdivided into: RH-SP-5d*,* RH-SP-10d. RH-SP-5d mice were treated for 5-days, and were euthanized at the end of the treatment, to be comparable to the corresponding non-treated subgroup (RH-NT-5d). RH-SP-10d mice were treated for 7-days, and were euthanized 10-dpi.

### Collection of Mice’ Blood Samples

The collected blood samples from different mice via cardiac puncture were left at room temperature for 30 minutes to allow complete coagulation. Aspirated sera were used for RNA extraction [27].

### Study of mmu-miR-511-5p Expression in the Sera of the Studied Groups by Reverse Transcription-Real Time Quantitative PCR (RT-qPCR) [23, 28]

#### RNA Extraction

Total RNA isolation was carried out using the miRNeasy Mini Kit (QIAGEN, Maryland, USA, Catalogue No. 217004) according to the manufacturer’s instructions. The final step involved eluting the extracted RNA using RNase free water.

##### RNA Quantitation and Quality Assessment

The concentration and purity of RNA were measured at wavelengths of 260, 280 and 230 nm using NanoDrop 2000 spectrophotometer. Samples with 260:230 ratio greater than 1.7 and a 260:280 ratio greater than 2.0 were included in the study.

##### Reverse Transcription (RT)

TaqMan^®^ MicroRNA Reverse Transcription Kit was used (ThermoFisher Scientific, catalogue number: 4366596) to convert RNA to complementary DNA (cDNA). The volumes shown in the supplementary section (a) were added to prepare the RT reaction master mix.

After preparing the master mix, the RT reaction was prepared by adding 7 μl master mix, 3 μl primer, and volume containing a concentration of 100 ng of extracted RNA. Nuclease free water was added to reach a total volume of 15-μl. Samples were applied to 25 thermal cycler which was programmed at 30 min hold at a temperature of 16°C, 30 min hold at a temperature of 42°C, 5 min hold at a temperature of 85°C, then lowering the temperature to 4°C and stopping the run [29]**.** Samples were chilled on ice for at least one min. cDNA was stored at − 20°C till used in real time qPCR.

##### Assay of miRNA (mmu-miR-511-5p) Expression Using Real Time qPCR

**Fig. 2:**
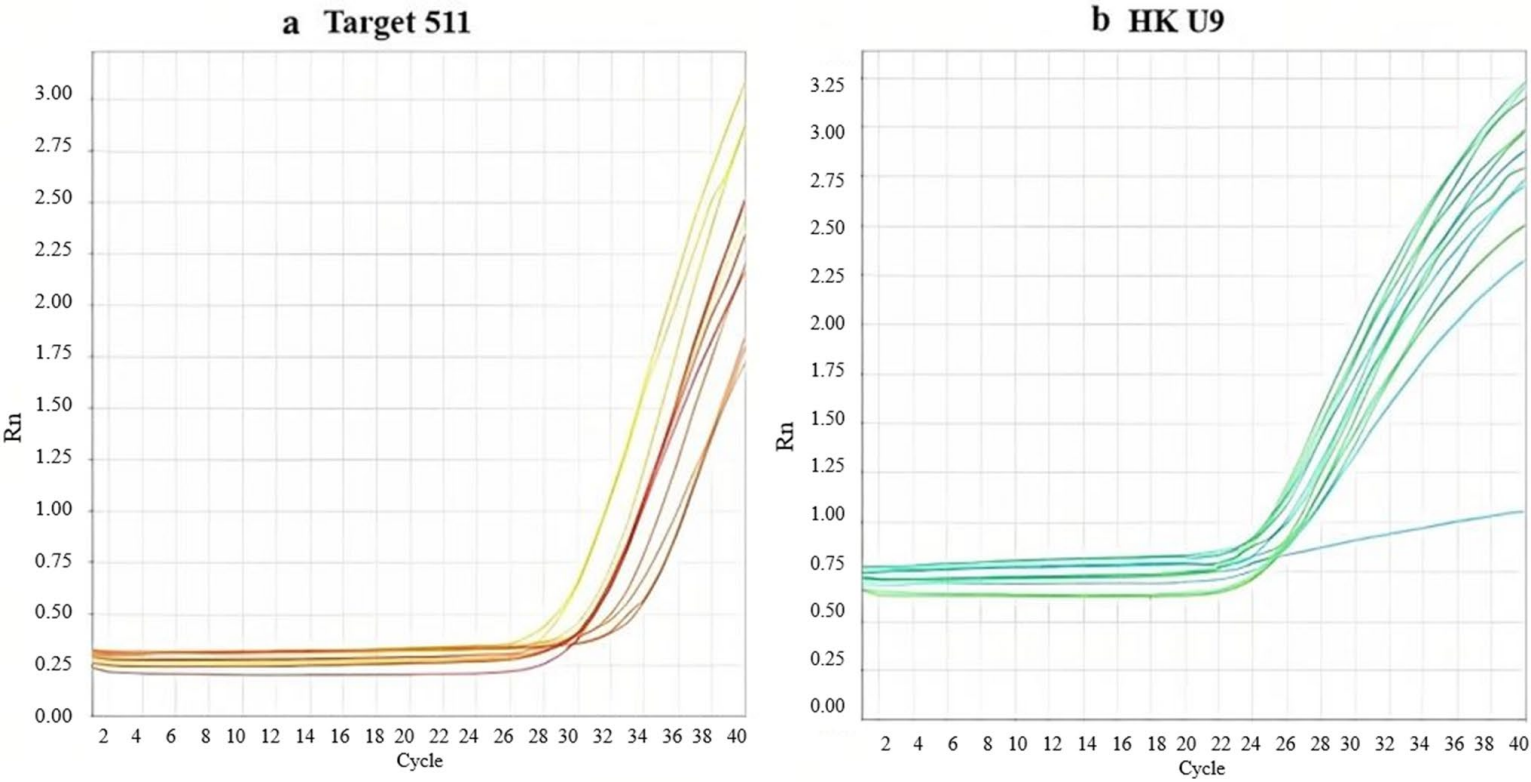
**a** Amplification plots of mmu-miR-511-5p; **b** Amplification plots of housekeeping gene (U6)

This was done using TaqMan MicroRNA Assay for both target miRNA (mmu-miR-511-5p, catalogue number A25576) and housekeeping gene (U6, catalogue number: 4427975).On the day of the assay, the reagents were thawed and mixed then the tubes were gently vortexed and briefly centrifuged to suspend the assays. TaqMan^®^ Universal Master was mixed by gently swirling the bottle. Reactions were performed in duplicates according to the manufacture’s recommendation. For each sample, the PCR reaction mix was prepared using the volumes shown in the supplementary section (b), where 20μL of PCR reaction mix were transferred to wells of the PCR strips. The plate was sealed and vortexed and the qPCR was programmed as follows: 1 cycle of enzyme activation for 10 min at temperature of 95°C, followed by 1 cycle of denaturation for 15 s at temperature of 95°C, then 40 cycles of annealing and extension for 60 s each at temperature of 60°C. Fluorescent signals from each sample were recorded at the endpoint of every cycle. Amplification plots of the studied miRNA and housekeeping gene were plotted and shown in Fig. 2 a, b

##### Analysis of miRNA Expression [30]

The fold change between a sample and a normal control for (mmu-miR-511-5p) was calculated with the relative quantification method (RQ = 2^−ΔΔCt^).

### Statistical Analysis

Data were fed to the computer and analyzed using IBM SPSS software package version 20.0. Qualitative data were described using number and percent. The Shapiro–Wilk test was used to verify the normality of distribution. Quantitative data were described using mean and standard deviation. Normally distributed quantitative variables were analysed using F-test (ANOVA) to compare between more than two groups, and Post Hoc test (Tukey) for pairwise comparisons. Student *t*-test was used for normally distributed quantitative variables to compare between two studied groups. Significance of the obtained results was judged at the 5% level.

## Results

**Fig. 3:**
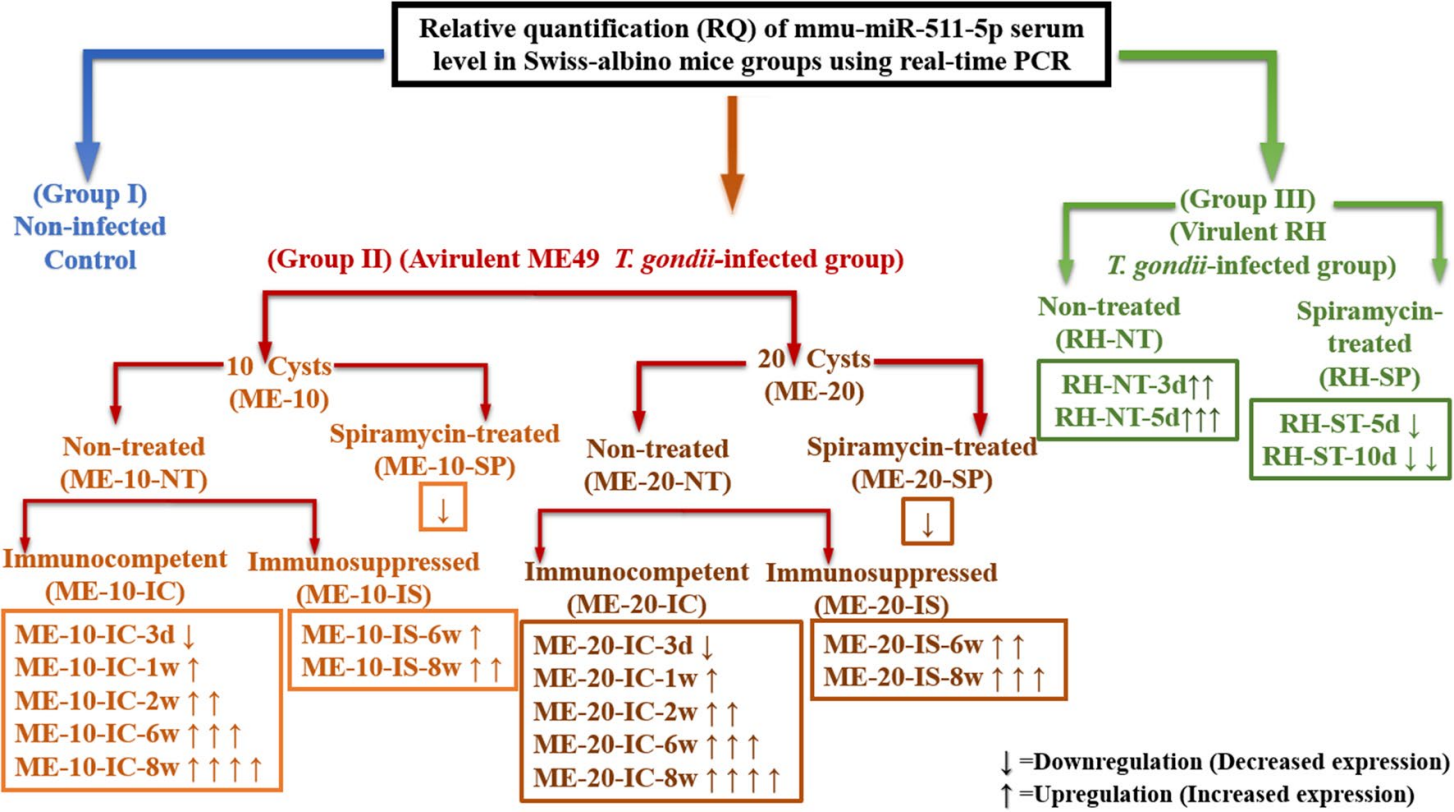
The mean relative quantification (RQ) of the serum level of microRNA mmu-miR-511-5p in the experimental groups

The mean relative quantification (RQ) of the serum level of microRNA mmu-miR-511-5p was demonstrated in Fig. 3, and was as follows:

### Group I (Non-Infected Group)

The RQ level was 0.0086 ± 0.0013, and this was referred to as a control.

### Group II (ME Group)

#### The Non-Treated Immunocompetent ME Subgroups

There was a non-significant decrease in the miRNA level in subgroup ME-10-IC-3d when compared to (group I). Yet, a significant upregulation was observed in subgroups; ME-10-IC-1w, ME-10-IC-2w, and ME-10-IC-6w. This increased expression reached the maximum 8-weeks PI (Table 1).

**Table 1:** Relative quantification (RQ) level of mmu-miR-511-5p in the sera of immunocompetent subgroups infected with 10 cysts (ME-10-IC) or 20 cysts (ME-20-IC) of the avirulent *T. gondii* strain (ME49)

		RQ level of mmu-miR-511-5P			
Non-infected control (Group I)					
Mean ± SD		0.0086 ± 0.0013			
		ME-10-IC	ME-20-IC	t	p_0_
Immunocompetent subgroups (ME Group)	3rd day			3.514^*^	0.017^*^
	Mean ± SD	0.0036 ± 0.0021	0.0006^a^ ± 0.0001		
	1st week			4.652^*^	0.003^*^
	Mean ± SD	0.0170^ab^ ± 0.0036	0.0242^ab^ ± 0.0012		
	2nd week			3.962^*^	0.003^*^
	Mean ± SD	0.0312^abc^ ± 0.0024	0.0364^abc^ ± 0.0021		
	6th week			2.613^*^	0.026^*^
	Mean ± SD	0.0741^abcd^ ± 0.0045	0.0826^abcd^ ± 0.0066		
	8th week			6.005^*^	< 0.001^*^
	Mean ± SD	0.1399^abcde^ ± 0.0039	0.1524^abcde^ ± 0.0033		
	F (p)	1645.217^*^ (< 0.001^*^)	1928.578^*^ (< 0.001^*^)		

ME-10: infected with 10 cysts of avirulent *Toxoplasma*/non-treated/immunocompetent group

ME-20: infected with 20 cysts of avirulent *Toxoplasma*/non-treated/immunocompetent group

*SD* Standard deviation, *RQ* relative quantification 6 replica for each group, ***t*** Student t-test (to compare between two groups euthanized groups at the same time), *F* F for ANOVA test, pairwise comparison between each 2 groups was done using Post Hoc Test (Tukey), *p* p value for comparing between the studied groups, *p*_*0*_ p value for comparing between 10 and 20 in each group

^*^ Statistically significant at p ≤ 0.05

^a^Significant with the non-infected control (group I) 3d: mice were euthanized three days PI

^b^Significant with 3d (ME-10-IC-3d/ME-20-IC-3d) in the same column 1w: mice were euthanized one-week PI

^c^Significant with 1w (ME-10-IC-1w/ME-20-IC-1w) in the same column 2w: mice were euthanized two weeks PI

^d^Significant with 2w (ME-10-IC-2w/ME-20-IC-2w) in the same column 6w: mice were euthanized six weeks PI

^e^Significant with 6w (ME-10-IC-6w/ME-20-IC-6w) in the same column 8w: mice were euthanized eight weeks PI

On the other hand, compared to the control, a significant downregulation was recorded in subgroup ME-20-IC-3d. Mice sacrificed later showed a significant upregulation, starting one-week PI, then, increased gradually during the 2nd and 6th weeks PI, reaching its highest level during the 8th weeks PI (Table 1).

The comparison between ME-10-IC and the corresponding ME-20-IC subgroups, revealed that, miRNA of ME-20-IC-3d was significantly lower than that of ME-10-IC-3d. Contrarily, the mean RQ level in mice infected with 20 cysts and euthanized during the 1st, 2nd, 6th and 8th weeks was significantly higher than that of the corresponding subgroups infected with 10 cysts and euthanized at the corresponding time points (Table 1).

#### The Non-Treated Immunosuppressed ME Subgroups

Compared to the control, a significant upregulation was observed in the immunosuppressed subgroups infected with 10 ME49 cysts; ME-10-IS-6w and ME-10-IS-8w. This was significantly higher than that of the corresponding immunocompetent subgroups (Table 2). A significant upregulation was also recorded in the immunosuppressed subgroups infected with 20 cysts; ME-20-IS-6w and ME-20-IS-8w. Moreover, these were significantly higher than the corresponding immunocompetent subgroups ME-20-IC-6w and ME-20-IC-8w (Table 2).

**Table 2:** Relative quantification (RQ) level of mmu-miR-511-5p in the sera of the immunocompetent subgroups and the corresponding immunosuppressed subgroups infected with 10 or 20 cysts of the avirulent *Toxoplasma gondii* strain (ME49)

Groups		RQ level of mmu-miR-511-5P			
Non-infected control (Group I)					
Mean ± SD		0.0086 ± 0.0013			
		ME-10-IC	ME-20-IC	t	p_0_
Immunocompetent subgroups	6 week (6w)				
	Mean ± SD	0.0741^a^ ± 0.0045	0.0826^a^ ± 0.0066	2.613^*^	0.026^*^
	8 week (8w)				
	Mean ± SD	0.1399^ab^ ± 0.0039	0.1524^ab^ ± 0.0033	6.005^*^	< 0.001^*^
		ME-10-IS	ME-20-IS		
Immunosuppressed subgroups	6 week (6w)				
	Mean ± SD	0.1437^ab^ ± 0.0481	0.2133^ab^ ± 0.0425	2.655^*^	0.024^*^
	8 week (8w)				
	Mean ± SD	0.2581^abcd^ ± 0.0710	0.4768^abcd^ ± 0.0770	5.112^*^	< 0.001^*^
	F (p)	34.937^*^ (< 0.001^*^)	123.763^*^ (< 0.001^*^)		

ME-10-IC: avirulent/10 cysts/non-treated/immunocompetent group. 6w: mice were euthanized six weeks PI

ME-20-IC: avirulent/20 cysts/non-treated/immunocompetent group. 8w: mice were euthanized eight weeks PI

ME-10-IS: avirulent/10 cysts/non-treated/immunosuppressed group

ME-20-IS: avirulent/20 cysts/non-treated/immunosuppressed group

*SD* Standard deviation 6 replica for each group, *t* Student t-test, *RQ* relative quantification, *F* F for ANOVA test, pairwise comparison between every comparable two groups was done using Post Hoc Test (Tukey), *p* p value for comparing between the studied groups, *p*_*0*_ p value for comparing between 10 and 20 in each group

^*^Statistically significant at p ≤ 0.05

^a^Significant with Non-infected control (group I)

^b^Significant with corresponding immunocompetent subgroup euthanized six weeks PI

^c^Significant with corresponding immunocompetent subgroup euthanized eight weeks PI

^d^Significant with corresponding immunosuppressed subgroup euthanized six weeks PI

Noteworthy that, the RQ level was significantly higher in the immunosuppressed subgroups infected with 20 cysts, than those infected with 10 cysts, and during the 8th week PI, than during the 6th week PI (Table 2).

#### Spiramycin-Treated ME Subgroups

The mean RQ level of ME-10-SP and ME-20-SP subgroups was significantly higher than that of the control. Yet, both showed a significant downregulation when compared to the corresponding non-treated subgroups; ME-10-IC-8w and ME-20-IC-8w (Table 3). There was non-significant difference between the treated subgroups infected with 10 and 20 cysts (Table 3).

**Table 3:** Relative quantification (RQ) level of mmu-miR-511-5p in the sera of spiramycin-treated subgroups and the corresponding non-treated subgroups infected with 10 or 20 cysts of the avirulent *Toxoplasma gondii* strain (ME49)

Groups	RQ level of mmu-miR-511-5P			
Non-infected control (Group I)				
Mean ± SD	0.0086 ± 0.0013			
Non-treated subgroups	ME-10-IC-8w	ME-20-IC-8w	t	p_0_
Mean ± SD	0.1399^a^ ± 0.0039	0.1524^a^ ± 0.0033	6.006^*^	< 0.001^*^
Spiramycin-treated subgroups	ME-10-SP	ME-20-SP	t	p_0_
Mean ± SD	0.0151^ab^ ± 0.0023	0.0190^ab^ ± 0.0084	1.113	0.310

Group I: non-infected control

ME-10-IC-8w: avirulent/10 cysts/non-treated/immunocompetent/euthanized eight weeks PI

ME-20-IC-8w: avirulent/20 cysts/non-treated/immunocompetent/euthanized eight weeks PI

ME-10-SP: avirulent/10 cysts/spiramycin-treated

ME-20-SP: avirulent/20 cysts/spiramycin-treated

*SD* Standard deviation 6 replica for each group, *t* Student t-test, *RQ* relative quantification, *p*_*0*_ p value for comparing between 10 and 20 in each group

^*^Statistically significant at p ≤ 0.05

^a^Significant with the non-infected control

^b^Significant with the corresponding non-treated, immunocompetent subgroup euthanized eight weeks PI

### Group III (RH Group)

Compared to the control, a significant upregulation was recorded in the non-treated subgroups as early as 3-dpi (RH-NT-3d). A further significant upregulation was evident 5-dpi (RH-NT-5d), compared to RH-NT-3d (Table 4).

**Table 4:** Relative quantification (RQ) level of mmu-miR-511-5p in the sera of group (III) mice infected with the virulent *Toxoplasma gondii* RH strain before and after treatment

	Non-infected control (Group I) (n = 6)	Non-treated subgroup (RH-NT)		Spiramycin-treated subgroup (RH-SP)	
		RH-NT-3d (n = 6)	RH-NT-5d (n = 6)	RH-SP-5d (n = 6)	RH-SP-10d (n = 6)
MiR-511-5p level					
Mean ± SD	0.0086 ± 0.0013	0.2699^a^ ± 0.0797	0.6649^ab^ ± 0.0654	0.0964^ad^ ± 0.0151	0.0105^c^ ± 0.0038
F (p)	209.560^*^ (< 0.001^*^)				

RH-NT-3d: virulent/non-treated/euthanized three days PI

RH-NT-5d: virulent/non-treated/euthanized five days PI

RH-SP-5d: virulent/spiramycin-treated/euthanized five days PI

RH-SP-10d: virulent/ spiramycin-treated/euthanized ten days PI

*SD* Standard deviation, *F* F for ANOVA test, pairwise comparison between each 2 groups was done using Post Hoc Test (Tukey), *p* p value for comparing between the studied groups

^*^Statistically significant at p ≤ 0.05

^a^Significant with the non-infected control

^b^Significance between RH-NT-3d and RH-NT-5d

^c^Significance between RH-SP-5d and RH-SP-10d

^d^Significance between RH-NT-5d and RH-SP-5d

Regarding the spiramycin-treated subgroups, the RQ level in RH-SP-5d was significantly lower than that of subgroup RH-NT-5d, although it was still significantly higher than that of the control. After the end of treatment, the level continued to decrease in RH-SP-10d, which was significantly lower than RH-SP-5d, yet, it was non-significant, compared to the control (Table 4).

## Discussion

The worldwide *T. gondii* infects a plenty of human populations, resulting in a wide range of symptoms and complications, that differ according to the host immune status [31–33].

Though serological techniques are counted on in diagnosis of toxoplasmosis, yet, they are deficient in early diagnosis and in follow-up of treatment. Therefore, novel sensitive method is urgently needed [6–8].

The detection of miRNAs in body fluids has raised the interest of their use as potential diagnostic biomarkers [34]. miR-511 is a short non-coding RNA, that was reported by Jia *et al*. (2014), to have a role as a potential biomarker for *T.gondii* diagnosis. However, being rarely investigated in the literature has motivated the current work authors to investigate its role during *T.gondii* infection [23].

The current results revealed that, the immunocompetent mice subgroups infected with 10 or 20 cysts of ME49 strain showed an upregulation in their mmu-miR-511-5p serum level, starting one-week PI, with a gradual significant increase, reaching its maximum 8-weeks PI. This could be explained by the progression of the infection and was in accordance to Jia *et al*. (2014) who found that, the circulating mmu-miR-511-5p has specifically increased in response to *T. gondii* infection [23].

This upregulation in miRNA level was also affected by the dose of infection, as evident by the significantly different increased expression among the subgroups infected with 10 cysts and corresponding subgroups infected with 20 cysts.

*Toxoplasma gondii* successful infection depends on the parasite ability to manipulate the host immune response, creating an environment that favors its survival and replication, through; balancing the pro- and anti-inflammatory cytokines, that control apoptosis and signaling pathways [35]. This is mediated via many mechanisms; one of them is manipulating the host miRNA, to establish favorable growth conditions, as reported by Zeiner *et al*. (2010) [36]. The authors reached a conclusion that, *Toxoplasma* infection has upregulated the levels of mature miR-17∼92-derived miRNAs in primary human cells. MiR-17∼92 cluster is crucial for apoptosis regulation, and accordingly, for *T. gondii* evasion [36].

Additionally, it was reported that, *T. gondii* causes abundant expression of mmu-miR-155-5p in murine immune cells involved in inflammatory responses, and an upregulation of mmu-miR-146a-5p, that is also involved in inflammation [37, 38]. The release of exosome-like vesicles containing several distinct miRNA species from cells infected with ME49 and RH strains was also reported, these miRNAs have immune regulatory functions [39].

Regarding mmu-miR-511-5p, the increased expression is multifactorial, as it has multiple predicted target genes, with many regulatory functions; involving immunological or pathological functions that can be affected by *T. gondii* infection with subsequent upregulation in its circulatory levels [40]. The miR-511 coding unit is located within the 5th intron of mannose receptor gene MRC1, that is expressed mainly in macrophages and dendritic cells and its expression regulates the expression of miR-511 [41].

The macrophages are known to be one of the main effector cells during the innate immune response against *T. gondii* infection [42]. Mosser and Edwards (2008) explored the full spectrum of macrophage activation and observed the macrophages’ remarkable plasticity that allows them to efficiently respond to the environmental signals and change their phenotype and physiology, that can be markedly altered by innate and adaptive immune responses. According to the stimulation type, macrophages can be either classically activated (M1) or alternatively activated (M2) [43]. The classically activated macrophage is activated through toll-like receptors (TLR) and interferon-γ (IFN-γ). It enhances killing of intracellular microorganisms, with increased cytokines’ secretion, and higher expression of co-stimulatory molecules. While the alternatively activated macrophage is activated by interleukin-4 (IL-4) or IL-13 and expresses IL-4 receptor-α, mannose receptor (CD206) and arginase-1 [43].

Later, Jensen *et al*. (2011) observed that different *Toxoplasma* strains could equally infect macrophages during the first three-days of intraperitoneal infection, with no differences in their recruitment [44]. These observations suggest that differences in parasite virulence might be partially due to how these strains interact with macrophages through effector molecules secreted by their rhoptries and dense granules that, accordingly, affect macrophage gene expression and control its polarization and activation to either M1 or M2.

In 2012, Hunter and Sibley, have identified a dense granule protein GRA15II, that is encoded by ME49 strain. This protein is secreted into the host cytosol, causing the activation of the classically activated macrophages (M1), while, ROP16 is secreted by RH strain and is responsible for the alternatively activated macrophages (M2) [42].

In addition to the aforementioned, Mazurek *et al*. (2022), outlined in their elegant paper [45], that, MiR-511-3p plays an important role in inflammation via the regulation of peroxisome proliferator-activated receptor γ (PPARγ) expression and Toll-like receptor 4 (TLR4) regulation in human dendritic cells (DCs). PPARγ is known to regulate many DC functions including pro-inflammatory cytokine production, migration, antigen presentation and activation. Downregulation of miR-511-3p increases PPARγ expression and leads to suppression of lipopolysaccharide-mediated inflammation (inhibition of NF-κB pathway). On the other hand, overexpression of miR-511-3p causes decreased activation of PPARγ and increased production of pro-inflammatory cytokines [46, 47]. This is consistent with the current findings where, miR-511 expression increased with progress of *Toxoplasma* burden and hence inflammation. It was also reported that Rho-associated coiled-coil containing protein kinase 2 (ROCK2), a serine/threonine kinase, is a direct target of miR-511-3p and promotes M2 polarization of macrophages by phosphorylating the interferon regulatory factor 4 transcription factor [48].

In 2015, Karo-Atar *et al*. showed that microRNA profiling of M1 and M2 macrophages revealed an opposing expression pattern for miR-511 [49]. Its level was downregulated in M1 which are activated in response to ME49 strain. On the other hand, miR-511 level was upregulated in M2 which are activated in response to RH strain. These can explain the current early downregulation of mmu-miR-511-5p recorded 3-dpi by the avirulent strain.

Contrarily, Jia *et al*. (2014) recorded upregulation in the plasma mmu-miR-511-5p level, 3-dpi [23], which can be attributed to the variation in the used mice strains, dose and route of infection between the current and the aforementioned study, as Jia *et al*. (2014) used female BALB/c mice, and individually, injected them intraperitoneally using 10^6^ tachyzoites. These variations may affect the pathological, the immunological outcomes, and accordingly, miRNA levels.

With the disease progression and activation of the adaptive immune response, other effectors may affect miRNA level. Researchers showed that within 8-days of oral ingestion of *Toxoplasma* tissue cysts, susceptible mice develop severe ileitis resulting in mucosal villus necrosis. This ileitis is due to the strong T-helper 1 biased immune response, characterized by the overproduction of pro-inflammatory mediators including; IFN-γ, TNFα, IL1β, IL18, and NO, commonly known as “cytokine storm” [50]. These cytokines in turn help in M1 polarization, leading to downregulation of miR-511-5p as reported by Curtale *et al*. (2017), who demonstrated a parallel inhibition of miR-511-5p expression in response to IFN-γ challenge [46]. The cytokine storm is controlled by the release of transforming growth factor β (TGF- β) by intraepithelial lymphocytes, and IL10 by CD4^+^ T cells. TGF- β and IL10 downregulate the IFN-γ response and inhibit chemokine production from enterocytes, limiting the recruitment of more immune cells, and thus, reducing inflammation [50]. In response to TGF-β release, miR-511-5p level is upregulated in monocytes, resulting in their desensitization due to downregulation of TLR4 present on their surface, which controls severe inflammation and dramatically reduce TNFα and other cytokines that are involved in ileitis [46]. The T_H_1 biased response, the cytokine storm, and its control by TGF- β and IL-10, all explain the present early downregulation, followed by upregulation in miR-511-5p levels in the macrophages and monocytes which is in turn reflected on its level in the host serum.

Tserel *et al*. (2011) found that miR-511 is important for the proper differentiation of monocyte-derived dendritic cells which are considered an important effector against toxoplasmosis [41]. Moreover, Puimège *et al*. (2015) reported that, MiR-511 upregulation is a mechanism of *T. gondii* immune evasion, through downregulation of the tumor necrosis factor receptor 1 protein (TNFR1) expression and subsequent TNF resistance which is an important effector during *Toxoplasma* infection [40].

Regarding the current immunosuppressed ME49 subgroups, immunosuppression was found to cause a significant upregulation of mmu-miR-511-5p level in mice euthanized 6- and 8-weeks PI, compared to the corresponding immunocompetent mice. This upregulation was also affected by the infective dose.

Qi *et al*. (2018) reported that cyclophosphamide increases T-helper 2 (T_H_2) cytokines (IL-4 and IL-10) [51] and meanwhile, it decreases T_H_1 cytokines, mainly; IFN-γ. These T_H_2 cytokines stimulate the expression of mmu-miR-511 in the macrophages [49], and thus, cause their polarization to M2 phenotype [44]. This provides an explanation of the current increase in miR-511 in the immunosuppressed subgroups. The reactivation of *Toxoplasma* infection which increases the parasite burden and the disease pathology after latency in response to immunosuppression augments miR-511 increase. These results suggest that, monitoring serum miR-511-5p levels can help in diagnosis of toxoplasmosis in immunosuppressed patients. In 2018, Mogahed *et al*. reported an upregulation of miR-712_3p level in the plasma of immunosuppressed Swiss-albino mice, infected with RH strain [21].

As regards spiramycin-treated ME subgroups, the level of mmu-miR-511-5p was downregulated after treatment. Although the downregulation was statistically significant when compared to the non-treated subgroup, yet the level did not reach that of the control. This decrease may be directly related to the decreased parasite burden, or indirectly affected by the immune cells’ response to treatment. Matsui *et al*. (2016) found that, spiramycin-treatment caused a decrease in T_H_2 cells [52]. The subsequent decrease in T_H_2 cytokines may explain the decrease in the miR-511 [49].

Concerning the RH group, the level of mmu-miR-511-5p in the sera of infected non-treated mice showed a significant increase, that was recorded as early as 3-days after infection, compared to the control. A further upregulation was recorded 5-dpi (the end of follow-up period). These results indicate that infection with the virulent strain has an early and up-regulatory effect on the circulating mmu-miR-511-5p. Similarly, in 2014, Jia *et al*. recorded an upregulation in mmu-miR-511-5p level in mice plasma 72-h after intraperitoneal infection with RH strain [23]. Mogahed *et al*. (2018) also recorded a significant up-regulation of plasma miR-712_3p, 3-and 5-dpi, in mice infected with RH strain [21].

The upregulation of mmu-miR-511-5p following infection with RH strain may be attributed to that: *T. gondii* virulent strain was found to produce rhoptry protein kinase (ROP16) [44], that can activate signal transducer and activator of transcription 6 (STAT6), leading to the induction of arginase, an enzyme that associates alternatively activated macrophages M2, leading to increased miR-511 expression inside the macrophages, and subsequently an upregulation of its serum level [42, 49, 53, 54].

The current spiramycin-treated RH subgroup showed a decrease in the serum miR-511 level, 5-dpi. This downregulation was significant, when compared to corresponding RH infected non-treated subgroup. The level continued to decrease until 10-dpi, when it reached a level that was non-significant when compared to the control. This post-treatment downregulation of miR-511 level may be related to the decrease in the parasite burden and amelioration of pathological changes in response to spiramycin treatment as proved by Omar *et al*. (2021) [55]. Spiramycin itself may affect the immune response, by decreasing the T-helper cells, leading to decrease in T_H_2 cytokines, with subsequent decrease in the miR-511 [49, 52].

The current results shed a light on the importance of resorting to alternative biomarkers for diagnosis of toxoplasmosis. Some of the advantages and challenges of using microRNAs in such a domain can be summarized as follows.

The easiness of obtaining a blood sample to assess the miRNA, the stability of miRNA in the blood even after repeated freeze–thaw cycles and meanwhile, its ability to reflect the tissue pathology, all make it an attractive marker. Looking at previous research on miRNA in the context of other infectious diseases [28], highlights some advantages and gives hope that this could be applied to toxoplasmosis. Advantages of miRNA biomarkers over the currently established techniques include; early detection, which is critical to prognosis and limitation of disease spread, hence giving a better chance of effective treatment. Expression of microRNAs may precede IgM class antibodies in parasitic diseases, since that IgM antibodies may eventually be undetected within the first weeks following infection [56].This is, especially, crucial during pregnancy, where early diagnosis and treatment of infected mothers decreases the risk of toxoplasmosis transmission to the foetus, thus, improving clinical outcomes [57]. MiRNAs as diagnostic biomarkers can also help in improved pathogen identification, especially when the symptoms of infectious diseases that first appear, are non-specific ones such as malaise, fever and headache [13]. Furthermore, microRNAs are biomarkers of high sensitivity and specificity for diagnosis of different stages of infection. According to Judice *et al*. (2016), other Apicomplexans such as; *Plasmodia* and *Cryptosporidium parvum* do not induce miR-511-5p expression [22], reinforcing its use as potential *T. gondii* biomarker. Besides, miRNA has an advantage over the current diagnostic tools, in differentiating new infections from old ones [13].

Unfortunately, a limitation would be that the same microRNA might be over or under expressed in various infections or health conditions. According to He *et al*. (2013), serum levels of several miRNAs that were suggested as biomarkers for some diseases might be dysregulated in other diseases [58]. Wang *et al*. (2019) b revealed that miR-511-5p was abnormally expressed in colorectal cancer [59].

Moving on to challenges, like any potentially new diagnostic biomarker, that is being translated into clinical practice, extensive research on humans has to be done, including large set of patients under different conditions of infection (as that was attempted to be done in the current research, using different strains, doses and immune status models). miR-511 levels in *Toxoplasma*-infected patients have to be compared to patients bearing other infections and inflammatory conditions. A cutoff value for miR-511 for *Toxoplasma* infection should be determined if possible, including different cutoffs for different disease stages. In addition, miR-511 expression levels should be thoroughly examined within the context of other microRNAs, so that a certain microRNA signature specific for *Toxoplasma* infection can reach consensus rather than a single microRNA, an era of what is called ‘Personalized Medicine’ [60].

## Conclusion

mmu-miR-511-5p could be a reliable biomarker for early diagnosis of infection with *T. gondii* ME49 strain, as early as one-week PI, and infection with the RH strain as early as 3-dpi. Its level is affected by the dose and progression of the infection. It can be also a promising diagnostic biomarker in the sera of immunosuppressed hosts, with the privilege of being less invasive than brain biopsy. Additionally, mmu-miR-511-5p serum level may be a useful indicator of successful treatment. So, it is a promising biomarker in immunocompetent, immunosuppressed, untreated and treated mice infected with ME49 or RH parasites.

To our knowledge, this is the first work assessing the expression of mmu-miR-511-5p in Swiss-albino mice sera using different infective doses of ME49 or RH *T. gondii* strains, in immunocompetent or immunosuppressed hosts, at different durations, before and after treatment with spiramycin.

### Supplementary Information

Below is the link to the electronic supplementary material.

Supplementary file1 (DOCX 12 KB)

Supplementary file2 (TIF 204 KB)

## Data Availability

Not applicable.
