# Supplementary material for: MicroRNA mmu-miR-511-5p: A promising Diagnostic Biomarker in Experimental Toxoplasmosis Using Different Strains and Infective Doses in Mice with Different Immune States Before and After Treatment
Source: Acta Parasitol. 2024 May 14;69(2):1253–66. doi: 10.1007/s11686-024-00851-w (PMC11182863; doi:10.1007/s11686-024-00851-w)
Supplement: Supplementary file 1 — Supplementary file1 (DOCX 12 KB) [file 11686_2024_851_MOESM1_ESM.docx]

**Supplementary Section**

Preparation of the RT reaction master mix; b Preparation of the PCR reaction mix
