# Supplementary figures and images for: MicroRNA mmu-miR-511-5p: A promising Diagnostic Biomarker in Experimental Toxoplasmosis Using Different Strains and Infective Doses in Mice with Different Immune States Before and After Treatment
Source: Acta Parasitol. 2024 May 14;69(2):1253–66. doi: 10.1007/s11686-024-00851-w (PMC11182863; doi:10.1007/s11686-024-00851-w)

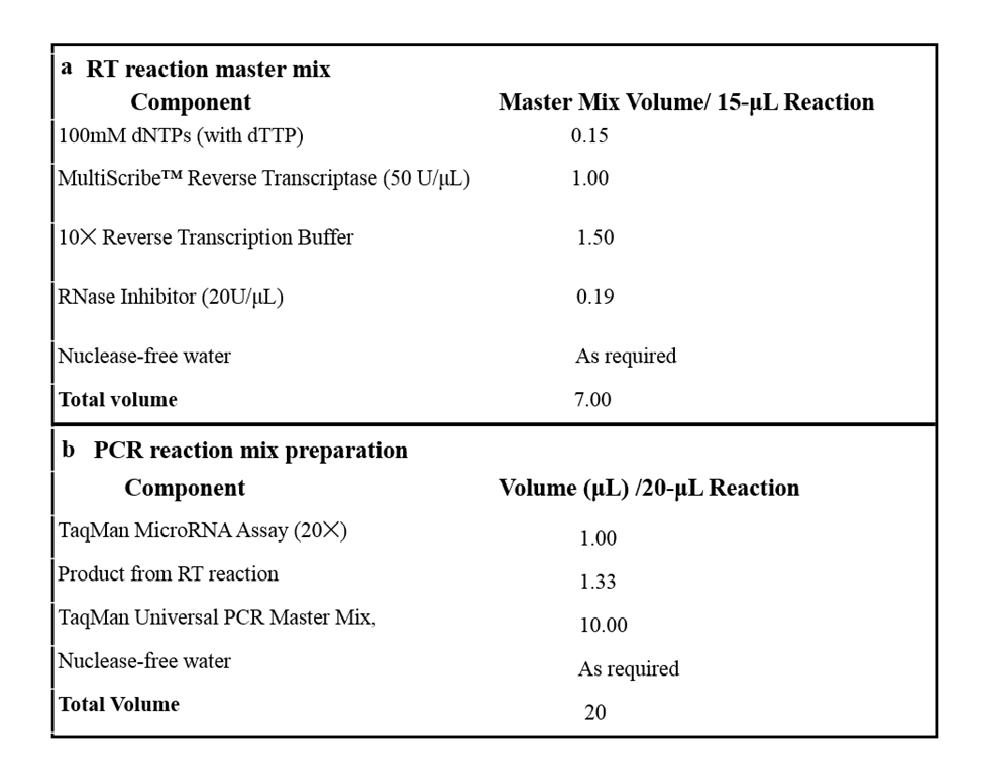

Supplement: Supplementary file 2 — Supplementary file2 (TIF 204 KB) [file 11686_2024_851_MOESM2_ESM.tif]
